# Event-based internet biosurveillance: relation to epidemiological observation

**DOI:** 10.1186/1742-7622-9-4

**Published:** 2012-06-18

**Authors:** Noele P Nelson, Li Yang, Aimee R Reilly, Jessica E Hardin, David M Hartley

**Affiliations:** 1 Department of Pediatrics, Georgetown University Medical Center, Washington, DC, 20007, USA; 2 Division of Integrated Biodefense, Georgetown University Medical Center, 2115 Wisconsin Ave, Suite 603, Washington, DC, 20007, USA; 3 Department of Microbiology and Immunology, Georgetown University Medical Center, Washington, DC, 20007, USA

**Keywords:** Biosurveillance, Infectious disease, Epidemiology, Disease-specific alerts, Internet media, Early warning, Pandemic (H1N1) 2009, Situational awareness, Outbreak detection

## Abstract

**Background:**

The World Health Organization (WHO) collects and publishes surveillance data and statistics for select diseases, but traditional methods of gathering such data are time and labor intensive. Event-based biosurveillance, which utilizes a variety of Internet sources, complements traditional surveillance. In this study we assess the reliability of Internet biosurveillance and evaluate disease-specific alert criteria against epidemiological data.

**Methods:**

We reviewed and compared WHO epidemiological data and Argus biosurveillance system data for pandemic (H1N1) 2009 (April 2009 – January 2010) from 8 regions and 122 countries to: identify reliable alert criteria among 15 Argus-defined categories; determine the degree of data correlation for disease progression; and assess timeliness of Internet information.

**Results:**

Argus generated a total of 1,580 unique alerts; 5 alert categories generated statistically significant (p < 0.05) correlations with WHO case count data; the sum of these 5 categories was highly correlated with WHO case data (r = 0.81, p < 0.0001), with expected differences observed among the 8 regions. Argus reported first confirmed cases on the same day as WHO for 21 of the first 64 countries reporting cases, and 1 to 16 days (average 1.5 days) ahead of WHO for 42 of those countries.

**Conclusion:**

Confirmed pandemic (H1N1) 2009 cases collected by Argus and WHO methods returned consistent results and confirmed the reliability and timeliness of Internet information. Disease-specific alert criteria provide situational awareness and may serve as proxy indicators to event progression and escalation in lieu of traditional surveillance data; alerts may identify early-warning indicators to another pandemic, preparing the public health community for disease events.

## Introduction

The World Health Organization (WHO) collects and publishes databases of statistics on confirmed and suspected disease outbreaks for select infectious diseases. The 2005 International Health Regulations (IHR), designed to ensure timely recognition of outbreaks of infectious disease with the potential to spread widely, requires WHO member nations to report outbreaks of international concern to the WHO within 24 hours of discovery [1-3]. Consistent with the IHR, during the initial months of the pandemic (H1N1) 2009 WHO requested that countries report the initial cases and thereafter the number of confirmed cases, and deaths in confirmed cases, for as long as feasible [4]. The WHO published weekly updates of pandemic (H1N1) 2009 case and fatality counts based on this reporting [5]. The resulting database represents one of the most comprehensive and timely outbreak reporting databases available to the public on the Internet.

Event-based biosurveillance, relying primarily on Internet sources, is a recognized approach to infectious disease outbreak detection. It complements traditional approaches to public health surveillance and can provide early warning of emerging events relative to such methods, where data may lag behind due to delays in sample collection, laboratory confirmation, and country reporting. There are several active event-based biosurveillance systems: Project Argus (Argus), Biocaster, Global Public Health Intelligence Network (GPHIN), HealthMap, MedISys, ProMED-mail (ProMED) and others [6,7]. Event reports are generated by automated machine-based processes for Biocaster, HealthMap and MedISys and written by human analysts or subject matter experts for Argus, GPHIN and ProMED. Manual report examination for relevancy typically occurs post-dissemination for the automated systems (e.g., do articles with the word “virus” in the title refer to a biological infection or an attack on computers?). With the exception of ProMed, which utilizes local observers on the ground for some of its outbreak reporting, event-based biosurveillance systems often disseminate reports that are not observer or laboratory verified (e.g., a cluster of unconfirmed human avian influenza cases in Vietnam). Thus the reports provide near real-time cueing and alerting to users, but they may lack specificity.

The specificity and timeliness of outbreak detection using event-based biosurveillance can be assessed by comparison with epidemiological data from official sources, such as WHO, when available. In general, detecting a new epidemic or outbreak (“signal”) amidst a varying background of disease (“noise”, e.g., normal seasonal influenza or influenza-like-illness) from the vast amount of information available on the Internet is difficult. Moreover, event-based biosurveillance systems can generate a sizable amount of information on any given outbreak topic, sometimes overwhelming users with specific interests. For example, Argus alone generated approximately 22,000 reports on pandemic (H1N1) 2009 from April 2009 to March 2010.

Establishing alert criteria can aid users in identifying relevant and anomalous events from such a large amount of information. Argus and other systems have established semi-automated (pushed via email) and customized (user created) email alerts as a method to improve signal detection, to notify users of emerging events of interest, and to allow for easier tracking of outbreaks or the aftermath of natural disasters.

However, establishing criteria for sending email alerts is complex. The WHO pandemic (H1N1) 2009 data provided a means to assess the timeliness of event-based biosurveillance in real-time and retrospectively, as well as to develop and evaluate alert criteria. In this study, a comparison of WHO epidemiological data and Argus reporting data was made in order to: 1) determine to what extent Argus alerting correlated with the epidemiological disease progression by country and region based on WHO data; 2) identify which alert criteria correlate the best with epidemiological data and provide the most reliable situational awareness; and 3) explore the timeliness of biosurveillance reporting.

## Methods

### Project Argus methodology

Project Argus, hosted at the Georgetown University Medical Center, is designed to report and monitor the evolution of biological events threatening human, plant and animal health globally, excluding the United States (US).[6-9] Argus collects, in an automated process, several thousand local, native-language Internet media articles daily.[10] Bayesian software tools and Boolean search strings, based on a taxonomy of infectious disease, identify candidate relevant articles. Regional experts, collectively fluent in roughly 40 languages, review these articles manually. Relevant media articles are identified based on direct indicators (reports of disease) and indirect indicators (socially disruptive events or precursors to disease, such as preventative measures or adverse enviro-climatic conditions). Regional experts write Argus reports based on these media articles; reports are posted to a password protected Internet portal for users to view.[11] Argus reported on pandemic (H1N1) 2009 from its identification in April 2009 to the post-pandemic period.[12]

### Comparing Argus alert data to WHO case counts

Argus employed email alerts to aid users in monitoring pandemic (H1N1) 2009 as it spread. Alert criteria were developed via an iterative process after assessing the progression of 2009 H1N1 between April 2009 – January 2010 and by monitoring WHO guidelines. Employed from August 2009 to January 2010 (see Table 1), email alerts were meant to capture increasing severity in a locale or region as portrayed in the media and were comprised of direct and indirect indicators[9] of disease. Senior staff reviewed Argus reports and reports meeting alert criteria were extracted. The report metadata and a link to the report on the Argus Internet portal were then emailed to users as a means of notification.

**Table 1:** Alert criteria for 2009 H1N1 pandemic general descriptions

**Alert**	**Description**	**Date of creation**
Alert 1	Large increase in case count (ie. resurgence, 50% increase in new cases from previous week)	November 5, 2009 (week 45)
Alert 2	Large increase in fatalities (ie. 50% increase in fatalities from previous week)	October 1, 2009 (week 40)
Alert 3	Confirmed cases or fatalities in healthcare workers, military personnel and/or national officials	July 7, 2009 (week 28)
	Cluster (>3) military personnel, health care workers and/or national officials	November 5, 2009 (week 45)
Alert 4	Fatalities in cases with no underlying health conditions	July 9, 2009 (week 28)
Alert 5	Severe manifestation, co-infection, re-infection (ie. encephalitis, atypical pneumonia, ICU admission with ventilator support, etc.)	July 30, 2009 (week 31)
Alert 6	Reports of overwhelmed ICUs and ventilator shortages as a result of 2009 – H1N1 Influenza infection	July 30, 2009 (week 31)
	Hospital/clinic infrastructure strain or collapse	October 1, 2009 (week 40)
Alert 7	Human-to-swine or swine-to-human transmission	July 9, 2009 (week 28)
Alert 8	Infection in species other than human or swine	July 7, 2009 (week 28)
Alert 9	Anti-viral resistance/failure; Non-traditional treatments	July 7, 2009 (week 28)
Alert 10	Virus mutation/reassortment	July 7, 2009 (week 28)
Alert 11	Vaccine failure, severe reaction, or black market sales	August 31, 2009 (week 36)
Alert 12	Massive release of antiviral or vaccine stockpile	July 7, 2009 (week 28)
Alert 13	Health policy change	July 9, 2009 (week 28)
Alert 14	Food safety (ie. economic loss due to suspected or feared food product contamination)	July 7, 2009 (week 28)
Alert 15	Border closures	July 9, 2009 (week 28)

The reporting alert criteria were reviewed and revised (weekly to monthly) during the course of the pandemic, based on reported feedback from system users. By the end of January 2010, 15 alert categories were in use. Some alert criteria were revised during the study period. For example, before November 2009 the number of healthcare workers, military personnel, and officials reported to be infected were recorded, but afterwards only clusters (> = 3) of cases for these categories were recorded. This change reflected the increased frequency of media reporting of individual cases over time and decreased value of monitoring individual case counts. Another example is that before October 2009 reports of overwhelmed ICUs and ventilator shortages were reported, but afterwards only overall hospital/clinic infrastructure strain or collapse was reported. The analysis for this study was performed based on the definition of the alert as specified during the time period of the analysis (Table 1).

The WHO 2009–2010 H1N1 country case count data was retrieved from the WHO website [5] on March 31, 2010. Argus timeliness could have also been assessed using the date of official confirmed case reporting from public Ministry of Health websites or the date of confirmed case reporting by countries to WHO where these sources of information were available. However, for this study we confined our analysis to WHO data only, because it provided comprehensive official information in one location. Argus weekly alert counts and WHO case counts for each alert category were recorded and plotted over time during a 6 month period from August 2009 to January 2010 (week 32, 2009 to week 5, 2010). The remainder of the analysis was performed during a 4 month period from October 2009 to January 2010 (week 41, 2009 to week 5, 2010) during which time 13 of the 15 alert criteria definitions were finalized. (Alert 1 was created and Alert 3 was modified during the first 4 weeks of the study period.) WHO weekly case updates were recorded by country. A time series of all Argus alert data combined was plotted against all WHO case counts from October 2009 to January 2010 (week 41, 2009 to week 5, 2010). The WHO case count and Argus alert counts were normalized between 0 and 1 prior to plotting as follows: count_j/Max{count_j}. Data were only used from countries covered by both WHO and Argus to provide an unbiased comparison (e.g. data from the US was not used since Argus does not cover the US).

A Pearson correlation matrix was generated for all Argus alerts to assess correlation with the data from WHO, the degree to which the alerts are related. Pearson correlation coefficients and corresponding p-values were generated for the combined alert data as well as for each alert individually in comparison to overall WHO case count data. A time series of alerts with WHO case data was plotted for all significant (p < 0.05) correlations.

Eight geographical regions were defined for purposes of our analysis: Africa; East Asia; Europe; Latin America and Caribbean; Middle East; Russia and Central Asia; South Asia; Southeast Asia, Oceania, and Canada. Nations in the WHO dataset were also assigned to these regions. Pearson correlation coefficients and corresponding p-values were generated for the total alert counts and WHO case counts by region.

All statistics were computed using R Version 2.11.0 [13].

### First confirmed pandemic (H1N1) 2009 case comparison

Reports of the first confirmed 2009 H1N1 influenza cases were recorded for Argus and/or WHO from April 24, 2009 to June 1, 2009. This time period was chosen due to the international focus on tracking pandemic (H1N1) 2009 cases spread in the initial months of the pandemic and the availability of daily pandemic (H1N1) 2009 WHO situation updates until June 1, 2009. The daily situation updates included WHO pandemic (H1N1) 2009 case data and were available on the WHO Global Alert and Response (GAR) website during this time period [14]. The Argus Internet portal was also monitored daily for media reports of confirmed cases in new countries. Argus timeliness was assessed using the date of first official confirmed case as reported by the WHO relative to the date of first case detected by Argus for a given country from Internet sources during the initial phases of the pandemic.

### Study period

**Figure 1:**
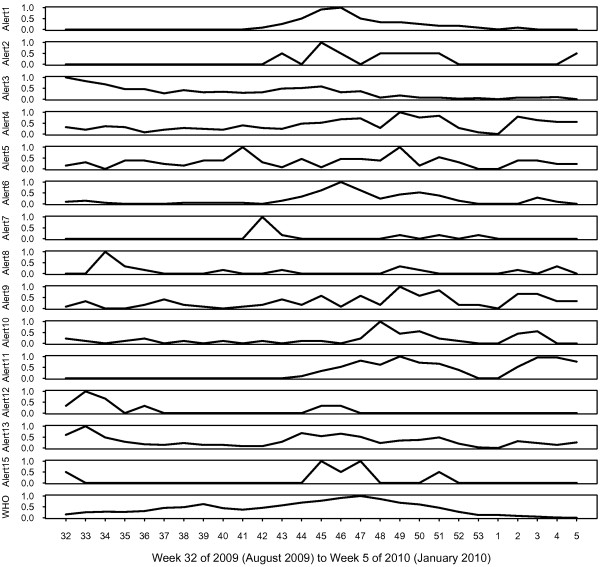
**Argus alerts and WHO case counts by week.** Y axes are all fixed to the same scale in this graph. Note that December 24^th^ 2009 to January 3^rd^ 2010 (week 52, 2009 to week 1, 2010) was a holiday period for Project Argus with substantially reduced reporting volume.

**Figure 2:**
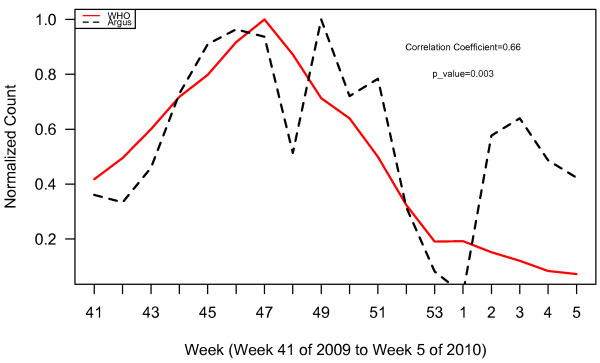
WHO normalized case counts compared to all Argus alerts pooled.

**Figure 3:**
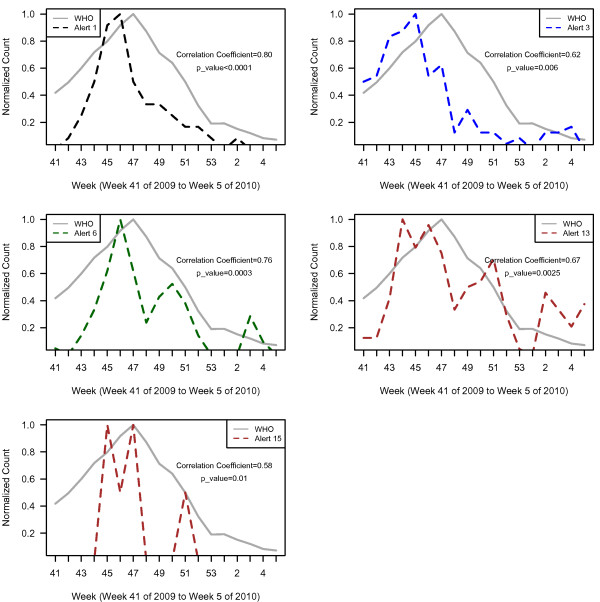
Plots of Argus alert 1 (large increase in case count), 3 (confirmed cases or fatalities in healthcare workers, military personnel and/or national officials), 6 (hospital/clinic infrastructure strain or collapse), 13 (health policy change) and 15 (border closures) versus WHO normalized case counts.

**Figure 4:**
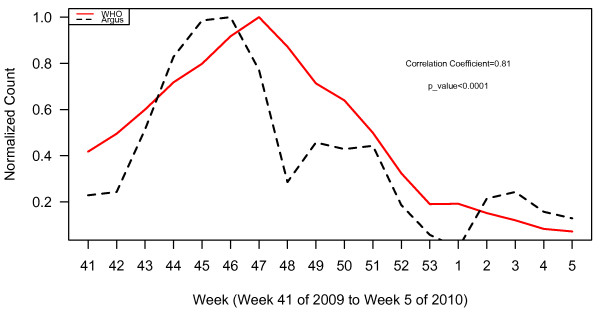
WHO normalized case counts compared to Argus alerts 1, 3, 6, 13 and 15 combined.

**Figure 5:**
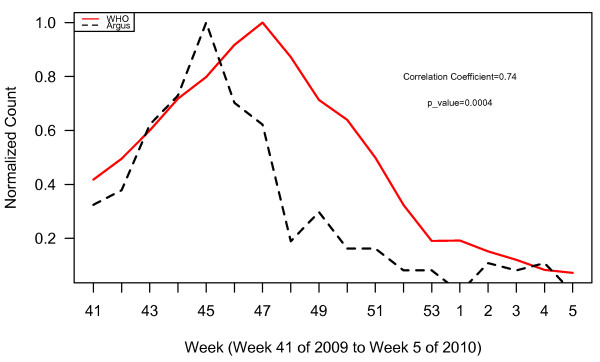
WHO normalized case counts compared to Argus alerts 1, 3, and 15 combined.

**Figure 6:**
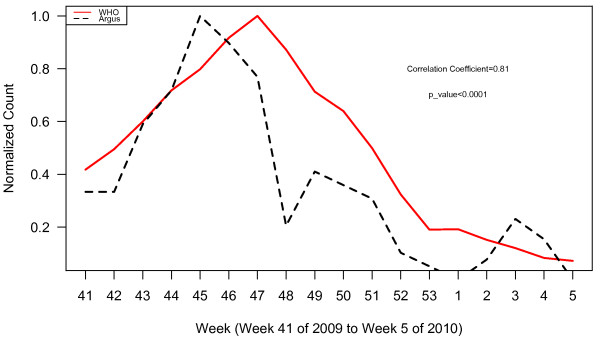
WHO normalized case counts compared to Argus alerts 3, 6, and 15 combined.

**Figure 7:**
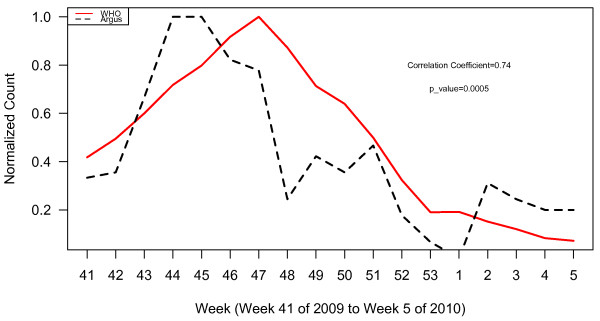
WHO normalized case counts compared to Argus alerts 3, 13 and 15 combined.

The overall study period was from April 2009 to January 2010. August 2009 to January 2010 is the time period used for the overall comparison of WHO case counts and Argus alert counts by week (Figure 1). October 2009 to January 2010 is the time period utilized for the comparison of Argus alerts compared to WHO case data after the alert criteria definitions were nearly finalized (Figures 2, 3, 4, 5, 6, 7, Table 2, Table 3). April 24, 2009 to June 1, 2009 is the study period utilized for the comparison of first confirmed cases for Argus and WHO (Table 4).

**Table 2:** Correlation coefficients for Argus alerts versus WHO case counts for October 2009 (week 41 2009) to January 2010 (week 5 2010)^*^

**Alert criteria as listed in Table **1	**Number of email alerts, N**	**Pearson correlation coefficient**	**P_value**
**Alert 1**	**56**	**0.80**	**<.0001**
Alert 2	9	0.46	0.0522
**Alert 3**	**147**	**0.62**	**0.0057**
Alert 4	228	0.24	0.3367
Alert 5	84	0.26	0.2979
**Alert 6**	**102**	**0.76**	**0.0003**
Alert 7	10	0.01	0.9651
Alert 8	7	0.12	0.6352
Alert 9	84	0.10	0.6878
Alert 10	35	0.25	0.3123
Alert 11	175	0.02	0.9312
Alert 12	2	0.43	0.072
**Alert 13**	**191**	**0.67**	**0.0025**
**Alert 15**	**6**	**0.58**	**0.0122**

*Statistically significant results (p < 0.05) are shown in bold.

**Table 3:** WHO case study comparison with Argus alerts by Region*

**Region**	**Alert**	**N**	**Pearson correlation coefficient**	**P-value**
Africa	Alert 3	8	0.59	0.0099
Africa	Alert 6	6	0.52	0.0254
Europe	Alert 1	12	0.61	0.0072
Europe	Alert 3	20	0.48	0.0446
Europe	Alert 5	13	0.60	0.0090
Europe	Alert 6	13	0.70	0.0012
Europe	Alert 11	46	0.53	0.0222
Europe	Alert 13	20	0.63	0.0047
Latin America	Alert 3	4	0.49	0.0391
Middle East	Alert 3	8	0.66	0.0027
Middle East	Alert 13	5	0.54	0.0212
Russia and Central Asia	Alert 6	22	0.56	0.0148
Russia and Central Asia	Alert 13	35	0.64	0.0040
South Asia	Alert 6	10	0.47	0.0487
Southeast Asia, Oceania, Canada	Alert 11	4	0.82	<0.0001

*Only statistically significant results shown (p < 0.05).

**Table 4:** **WHO/Argus 1**^**st**^** date of confirmed 2009 H1N1 case by country**

**Country count**	**Country**	**Argus Date of 1st confirmed 2009 H1N1 case (2009)**	**WHO Date of 1st confirmed 2009 H1N1 case (2009)**	**Delta Argus Date compared to WHO (Argus date minus WHO date)**	**URL identified by Argus**^**a**^
1	Argentina	May 8	May 9	1	http://www.surenio.com.ar/index.php?s=ARgmunqs$$diarios/veo$W082qf5qrwtk2y8siekftf
2	Australia	May 9	May 9	0	http://www.brisbanetimes.com.au/national/first-swine-flu-case-confirmed-in-qld-20090509-aybo.html
3	Austria	Apr 29	Apr 29	0	http://www.oe24.at/oesterreich/chronik/oberoesterreich/Zwei_neue_Verdachtsfaelle_in_Oe_0457579.ece
4^b^	Bahamas	[Jun 3]	Jun 1	[−2]	http://www.caribbeannetnews.com/bahamas/bahamas.php?news_id=16857&start=0&category_id=25
5	Bahrain	May 26	May 27	1	http://www.almotamar.net/news/70382.htm
6	Belgium	May 13	May 15	2	http://www.7sur7.be/7s7/fr/1502/Belgique/article/detail/853113/2009/05/13/Un-cas-de-grippe-mexicaine-confirm-en-Belgique.dhtml
7	Bolivia	May 29	Jun 1	3	http://www.lostiempos.com/diario/actualidad/tragaluz/20090529/alerta-en-el-pais-por-dos-casos-positivos-de-gripe_11560_18658.html
8	Brazil	May 8	May 8	0	http://oglobo.globo.com/economia/mat/2009/05/08/saude-confirma-primeiros-4-casos-de-gripe-suina-no-brasil-755767939.asp
9	Canada	Apr 26	Apr 27	1	http://www.theprovince.com/Health/Vancouver+have+swine+after+trip+Mexico/1536270/story.html
10	Chile	May 15	May 18	3	http://www.emol.com/noticias/nacional/detalle/detallenoticias.asp?idnoticia=358197
11	China	May 11	May 11	0	http://news.yninfo.com/china/gdxw/200905/t20090511_803749.htm
12	Colombia	May 3	May 4	1	http://timesofindia.indiatimes.com/World/Colombia-confirms-1st-swine-flu-case/articleshow/4479277.cms
13	Costa Rica	Apr 28	May 2	4	http://www.nacion.com/ln_ee/2009/abril/28/pais1948097.html
14	Cuba	May 12	May 13	1	http://www.cubaencuentro.com/es/cuba/noticias/el-gobierno-confirma-el-primer-caso-en-un-estudiante-mexicano-177458#comment
15	Cyprus	May 30	Jun 1	2	http://www.europapress.es/internacional/noticia-gripe-chipre-confirma-primer-caso-nueva-gripe-20090530181929.html
16	Czech Republic	May 26	May 29	3	http://www.ceskenoviny.cz/praseci-chripka/zpravy/v-cr-se-objevil-prvni-pripad-nove-chripky/378741
17	Denmark	May 1	May 1	0	http://nyhederne.tv2.dk/article.php/id-22072069.html
18	Dominican Republic	May 27	May 29	2	http://www.almomento.net/news/127/ARTICLE/34416/2009-05-27.html
19	Ecuador	May 15	May 16	1	http://www.lefigaro.fr/flash-actu/2009/05/15/01011-20090515FILWWW00494-grippe-a-1er-cas-en-equateur.php
20^b^	Egypt	May 18,			http://www.almesryoon.com/ShowDetails.asp?NewID=64086&Page=1
		[Jun 2]	[Jun 3]	16,1	http://www.alyoum7.com/News.asp?NewsID=104988
21	El Salvador	May 4	May 4	0	http://www.diariocolatino.com/es/20090504/nacionales/66409/
22	Estonia	May 29	Jun 1	3	http://rus.postimees.ee/?id=125606
23	Finland	May 12	May 14	2	http://www.thl.fi/sv_SE/web/sv/meddelande?id=13260
24	France	May 1	May 2	1	http://www.leparisien.fr/societe/exclusif-polemique-sur-le-premier-cas-avere-de-grippe-a-en-france-01-05-2009-498603.php
25	Germany	May 1	May 1	0	http://www.sueddeutsche.de/wissen/526/467104/text/
26	Greece	May 18	May 20	2	http://www.madata.gr/index.php/diafora/health/36135.html
27	Guatemala	May 6	May 6	0	http://www.laprensa.com.ni/archivo/2009/mayo/05/noticias/ultimahora/325718.shtml
28	Honduras	May 22	May 25	3	http://www.latribuna.hn/news/45/ARTICLE/64704/2009-05-22.html
29	Hong Kong	May 1	May 1	0	http://home.kyodo.co.jp/modules/fstStory/index.php?storyid=436705
30	Hungary	May 29	Jun 1	3	http://www.alertnet.org/thenews/newsdesk/LT682796.htm
31	Iceland	May 24	May 25	1	http://www.visir.is
32	India	May 12	May 17	5	http://news.xinhuanet.com/english/2009-04/28/content_11271565.htm
33	Ireland	May 3	May 3	0	http://c.moreover.com/click/here.pl?r1957191313
34	Israel	Apr 28	Apr 28	0	http://www.jpost.com/servlet/Satellite?cid=1239710811758&pagename=JPost%2FJPArticle%2FPrinter
35	Italy	May 2	May 3	1	http://www.almanar.com.lb/NewsSite/NewsDetails.aspx?id=84034&language=en
36	Jamaica	May 31	Jun 1	1	http://flutrackers.com/forum/showthread.php?t=107518
37	Japan	May 9	May 9	0	http://story.malaysiasun.com/index.php/ct/9/cid/ed68ecccb9e5520c/id/24299905/
38	Kuwait	May 24	May 25	1	http://www.7days.ae/storydetails.php?id=78470&title=Swine%20flu%20confirmed%20in%20Gulf
39	Malaysia	May 15	May 17	2	http://thestar.com.my/news/story.asp?file=/2009/5/15/apworld/20090515144227&sec=apworld
40	Mexico	Apr 24	Apr 24	0	http://www.oem.com.mx/elsoldetampico/notas/n1136513.htm
41	Netherlands	Apr 30	Apr 30	0	http://www.radionetherlands.nl/news/zijlijn/6280929/Netherlands-reports-first-case-of-Mexican-flu
42	New Zealand	Apr 28	Apr 28	0	http://www.rosbalt.ru/2009/04/28/636894.html
43	Norway	May 10	May 11	1	http://www.straitstimes.com/Breaking%2BNews/World/Story/STIStory_374853.html
44	Panama	May 8	May 9	1	http://www.interfax.ru/news.asp?id=78749
45	Paraguay	May 29	Jun 1	3	http://podii.com.ua/world/2009/05/29/144209.html
46	Peru	May 15	May 16	1	http://www.huaralenlinea.com/noticias/se-confirmo-el-primer-caso-de-gripe-ah1n1-en-el-peru/
47	Philippines	May 21	May 22	1	http://www.gmanews.tv/story/162338/RP-confirms-first-case-of-A(H1N1)-flu-virus-infection
48	Poland	May 6	May 7	1	http://fakty.interia.pl/news/swinska-grypa-na-podkarpaciu,1302142?source=rss
49	Portugal	May 4	May 4	0	http://diariodigital.sapo.pt/news.asp?section_id=62&id_news=386022
50	Romania	May 27	May 29	2	http://www.infox.ru/03/body/2009/05/27/Svinoy_gripp_tyepyer.phtml
51	Russia	May 22	May 23	1	http://medportal.ru/mednovosti/news/2009/05/22/gripp/
52	Singapore	May 27	May 27	0	http://www.moh.gov.sg/mohcorp/pressreleases.aspx?id=21914
53	Slovakia	May 28	May 29	1	http://www.zzz.sk/?clanok=6408
54	South Korea	May 2	May 3	1	http://c.moreover.com/click/here.pl?r1955932337
55	Spain	Apr 27	Apr 27	0	http://www.leparisien.fr/societe/espagne-premier-cas-de-grippe-porcine-en-europe-27-04-2009-494039.php
56	Sweden	May 6	May 6	0	http://www.smittskyddsinstitutet.se/presstjanst/pressmeddelanden-och-pressinbjudningar/2009/det-forsta-bekraftade-fallet-i-sverige-av-den-nya-influensan/
57	Switzerland	Apr 30	Apr 30	0	http://www.lematin.ch/actu/suisse/premier-cas-avere-suisse-115951
58	Thailand	May 12	May 13	1	http://www.thailandoutlook.tv/toc/ViewData.aspx?DataID=1014540
59	Turkey	May 15	May 17	2	http://news.bakililar.az/news_svinoy_qripp_dobralsya_22165.html
60^b^	United Arab Emirates	May 25	[June 8]	[14]	http://www.tradearabia.com/news/HEAL_161850.html
61	United Kingdom	Apr 27	Apr 28	1	http://www.google.com/hostednews/ukpress/article/ALeqM5g8Ca5Zzy_P8WjU4vvWx3NbcbU10w
62	Uruguay	May 28	May 29	1	http://www.extra.ec/noticias.asp?codigo=20090527143033
63	Venezuela	May 29	Jun 1	3	http://www.el-carabobeno.com/p_pag_not.aspx?art=a290509e07&id=t290509-e07
64	Vietnam	May 31	Jun 1	1	http://www.siasat.com/english/index.php?option=content&task=view&id=343158&Itemid=&cattitle=World

^a^Note that the URL may not be currently active.

^b^Date is outside the study period for first cases (April 24^th^ to June 1^st^).

## Results

### Alert data compared to WHO case counts

Using the alert criteria in Table 1, a total of 1,580 alerts were recorded from 1,499 Argus reports covering 122 countries in the 6 month time period of August 2009 to January 2010 (week 32, 2009 to week 5, 2010). Note that multiple alerts were generated for some Argus reports. WHO recorded 244,196 pandemic (H1N1) 2009 cases during this time period (Figure 1).

Plots of WHO case data and all Argus alerts pooled from October 2009 to January 2010 (week 41, 2009 to week 5, 2010) appear in Figure 2. During this time period, alert categories 1 (i.e. increase in case counts), 3 (i.e. clusters of cases or fatalities of health care workers, military personnel or national officials), 6 (i.e. healthcare facility strain or collapse), 13 (i.e. health policy change) and 15 (i.e. border closure) generated statistically significant (p < 0.05) correlations with WHO case count data with correlation coefficients (r) of 0.80, 0.62, 0.76, 0.67, and 0.58 respectively (Table 2). The time series of the sum of alerts 1, 3, 6, 13 and 15 was highly correlated with WHO case data (r = 0.81, p < 0.0001). The plots for these alerts are illustrated in Figures 3 and 4. Argus alerts 1, 6 and 13 are significantly correlated (alerts 1 and 6: r = 0.87, p < 0.0001; alerts 1 and 13: r=0.80, p<0.0001), alerts 6 and 13: r = 0.81, p < 0.0001). Figures 5, 6, 7 illustrate the combined plots with alerts 3, 15 and 1 or 6 or 13. No reports met the alert 14 (food safety) criteria.

Data was present in both WHO and Argus for 49 countries in the 8 regions. Data from Germany, Portugal, Canada, and Brazil was not available on the WHO website as of March 31, 2010. Alert time series with significant correlation to WHO case data were 1, 3, 5 (i.e. severe manifestation, co-infection or re-infection), 6, 11 (i.e. vaccine failure, severe reaction, or black market sales) and 13 (Table 3).

### First confirmed pandemic (H1N1) 2009 case comparison

Argus and WHO collectively identified 64 countries with confirmed cases of pandemic (H1N1) 2009 from May 8, 2009 to June 1, 2009 (Table 4). Argus reported the first confirmed case on the same day as WHO for 21 of the 64 countries. Argus reported from 1 to 16 days ahead of WHO for 42 countries: 1 day ahead for 22 countries; 2 days ahead for 8 countries; 3 days ahead for 8 countries, 4 days ahead for 1 country (Costa Rica) and 5 days ahead for 1 country (India). Two countries were identified by Argus only during the study period. Egypt was identified by WHO on June 3 and United Arab Emirates was identified by WHO on June 8, 16 days and 14 days after Argus, respectively. One country was identified by WHO only during the study period, Bahamas, and was reported by Argus 2 days later. Note that the first case in Egypt was identified by Argus on May 18, but did not appear in the sources monitored again until after the study period on June 2. Both dates are recorded in Table 4.

## Discussion

As the media coverage intensifies during the course of a high profile event, such as pandemic (H1N1) 2009, establishing alert criteria can help guide users of Internet based biosurveillance systems. In this study, alert categories 1 (i.e. increase in case counts), 3 (i.e. cases or fatalities of health care workers, military personnel and/or national officials), 6 (i.e. healthcare facility strain or collapse), 13 (i.e. health policy change) and 15 (i.e. border closure) were significantly correlated with the WHO confirmed case count in the four month study period (October 2009 to January 2010 (week 41, 2009 to week 5, 2010). Thus, alerts targeting direct indicators (Alerts 1 and 3) and indirect indicators (Alerts 6, 13, and 15) provided situational awareness during the pandemic.

Increase in case counts (Alert 1) is the most similar alert category to WHO case count data. The significant correlation suggests that reports of confirmed cases in the media are consistent with confirmed cases identified through public health surveillance and testing. A rising number of cases or fatalities of health care workers, military personnel or national officials (Alert 3), who are often more aware of prevention measures than the general public, is an indication of an emerging or escalating infectious disease outbreak, consistent with a rise in case counts. Health care facility strain or collapse (Alert 6) is an indirect indicator of increasing case counts and/or increase in health care worker cases or fatalities.

Though only six alerts were generated for border closure (Alert 15), it is not surprising that the alert is correlated with WHO case data considering the severity of an event that would warrant such an action. Similarly, massive release of anti-virals or vaccine stockpiles (Alert 12) indicates a severe escalation or perceived escalation in cases or deaths. This alert did not reach significance, however, likely because only two alerts were generated for this category. Alerts 1, 6 and 13 are also correlated with each other and maintain a highly significant correlation with WHO case counts when compared individually to WHO case counts along with alerts 3 and 15. An increase in case counts would lead to healthcare infrastructure strain and health policy change, likely accounting for the intra-alert correlation. Comparison of alerts in pandemic versus non-pandemic years is required for verification; however, this study suggests that Alerts 1, 3, 6, 13 and 15 may all serve as proxy indicators in the media of an emerging or escalating event on the ground and could serve as surveillance measures in conjunction with public health surveillance for a future pandemic.

The other alerts may not have been significantly correlated with WHO case counts due to the relatively mild manifestation of the pandemic (H1N1) 2009 without a virulent secondary wave or changes in transmission patterns.[15] Though reports of atypical clinical manifestations, transmission to other species, anti-viral resistance[16] and failure and viral mutations were prevalent in the media, such mechanisms appear to have not contributed to a significant escalation in case count.[15] These alerts, however, could serve as potential indicators for a future pandemic. A large increase in fatalities (Alert 2) was borderline significant with only 9 alerts generated. Again this is likely due to the mild nature of the pandemic, with an estimated 12,000 deaths, compared to previous pandemics, 1918, 1957 or 1968, with estimated attributable mortality of 50 to 100 million, 1–2 million, and 1 million, respectively.[15,17,18]

Correlation analysis by region showed some variation in the significant alerts as was expected based on the differences in severity of the pandemic, capacity for disease detection and capability for response for each region. Alert 5 (i.e. severe manifestation, co-infection or re-infection) and alert 11 (i.e. vaccine failure, severe reaction, or black market sales) emerged as significantly correlated to WHO case counts in Europe and in Europe, South Asia-Canada-Oceana, respectively, though they were not significant when global WHO case counts were considered. These results suggest that regional differences in the evolution of the pandemic are important to consider when developing alert criteria. Alerts 1, 3, 6 and 13 were each significant in one or more regions, which further supports their appropriateness for global surveillance.

Utilizing Internet media sources, Argus identified the first cases of confirmed pandemic (H1N1) 2009 published on the Internet an average of 1.5 days ahead of WHO official reporting (range 1 to 16 days) for all 64 non-US countries reporting by June 1, 2009. This was expected since information from Internet media reports is often timelier than the official reporting of cases to the public after laboratory confirmation. Though in this case the lead-time may be only a few days, this study provides evidence of the validity of using event-based biosurveillance for detecting emerging biological events.

This study had limitations. The alert criteria evolved from initiation in August 2009 through November 2009. However, the study period chosen for the majority of the analysis was after October in order to mitigate any bias from changing alert criteria. In addition, the alert criteria changes were small, geared toward making the alert criteria more specific and did not significantly impact the results (data not shown). In event-based biosurveillance studies there is often a lack of robust gold standard official comparison data. WHO data can be limited by delays in country reporting and under-reporting, however for the 2009 pandemic WHO was considered a timely and accurate source of global data [19]. Finally, the study had a restricted time window. Fears of a virulent resurgence of the virus in a second wave were unfounded and when WHO case counts and Argus alerts decreased to low levels in January 2010, the study was ended. Nonetheless, sufficient data was collected to identify significant indicators of the evolving pandemic.

The pandemic (H1N1) 2009 was of global significance and a main focus of local, national and international public health organizations, particularly during the initial phase. However, there are numerous human, animal and plant diseases that are economically important but are not normally tracked by public health organizations, suggesting that Internet surveillance of such diseases could provide lead-time of an outbreak compared to traditional methods [20]. When surveillance for indirect indicators (suspected cases or prevention measures) is performed in addition to direct reports of disease, the lead-time often increases further.[8,21] Surveillance of pandemic (H1N1) 2009 serves as an example of the real-time capability of identifying emerging disease events in general, particularly events that may be evident in local media in the regional vernacular.

Other event-based biosurveillance systems have demonstrated the effectiveness of extracting relevant information from Internet media sources as a means for detecting and monitoring disease events.[21] Internet media reporting provides an emerging resource for early detection of new events and for providing situational awareness of evolving events, particularly when official sources may not be available. Alerts based on media reports can provide event situational awareness and cue users of shifts in infectious disease trends. As the number of online news media sources, including social media sources with user-generated content, continues to expand, event-based biosurveillance will play an increasingly important role in disease surveillance. On-going validation and verification of event-based biosurveillance methods with epidemiological and clinical data by users and surveillance system developers will increase the robustness of this approach for detecting and tracking emerging events.

## Competing interests

The authors have no financial or non-financial competing interests to declare.

## Authors’ contributions

NN drafted the manuscript and co-conceived of the research topic. LY performed the statistical analysis and helped to draft the manuscript. AR participated in the design of the study and helped draft the manuscript. JH participated in the design of the study and helped draft the manuscript. DH drafted the manuscript and co-conceived of the research topic. All authors read and approved the final manuscript.

## Authors' information

NN holds a MD in International Health and PhD in Epidemiology and is a Research Assistant Professor in the department of Pediatrics at Georgetown University Medical Center. LY is a biostatistician and holds a Masters degree in epidemiology and biostatistics. AR holds a Masters degree in Microbiology and Immunology; she is also an ISDS member and has worked for over four years in the biosurveillance field. JH holds a Masters degree in Emerging Infectious Diseases and Biohazardous Threat Agents and has worked over four years in the biosurveillance field. DH is Research Associate Professor in the department of Microbiology and Immunology at Georgetown University Medical Center; his research applies mathematical modeling and related methodologies to understand the dynamics of disease in human and animal populations.

## References

[B1] World Health Organization International Health Regulations (2005) 2008 2 Geneva: The Organization [cited 2009 May 21]. http://whqlibdoc.who.int/publications/2008/9789241580410_eng.pdf

[B2] Baker MG Forsyth AM The new International Health Regulations: a revolutionary change in global health security N Z Med J 2007 Dec 14 120 1267 U2872 18157198

[B3] Wilson K McDougall C Forster A The responsibility of healthcare institutions to protect global health security Healthc Q 2008 12 1 56 60 19142064 10.12927/hcq.2009.20415

[B4] World Health Organization Global Alert Response. Human infection with pandemic (H1N1) 2009 virus: updated interim WHO guidance on global surveillance 10 July 2009 (originally posted on 29 April 2009). http://www.who.int/csr/resources/publications/swineflu/interim_guidance/en/index.html

[B5] WHO FluNethttp://apps.who.int/globalatlas/

[B6] Hartley DM Nelson NP Walters R Arthur R Yangarber R Madoff L The landscape of international event-based biosurveillance Emerging Health Threats Journal 2009 3 e3 http://www.eht-forum.org/JournalStore/Journal 10.3134/ehtj.10.003 22460393 PMC3167659

[B7] Walters R Harlan P Nelson NP Hartley DM Voeller J Data sources for biosurveillance Wiley handbook of science and technology for homeland security 2010 New York: John Wiley & Sons 1 17

[B8] Nelson NP Brownstein JS Hartley DM Event-based biosurveillance of respiratory disease in Mexico, 2007–2009: connection to the 2009 influenza A(H1N1) pandemic? Euro Surveill 2010 15 30 19626 http://www.eurosurveillance.org/ViewArticle.aspx?ArticleId=19626 20684815

[B9] Wilson JM Polyak MG Blake JW Collmann J A heuristic indication and warning staging model for detection and assessment of biological events JAMIA 2008 15 158 171 18096906 10.1197/jamia.M2558 PMC2274782

[B10] Jeff Collmann Adam Robinson Zeng D, Chen H, Castillo-Chavez C, Lober WB, Thurmond M Designing Ethical Practice in Biosurveillance The Project Argus Doctrine Infectious Disease Informatics and Biosurveillance, series: Integrated Series in Information Systems, Volume 27 2011 New York: Springer 23 44 http://www.springer.com/public+health/book/978-1-4419-6891-3

[B11] Open Source Center http://www.opensource.gov

[B12] Centers for Disease Control and Prevention (CDC) Swine influenza A (H1N1) infection in two children--southern California, March-April 2009 MMWR Morb Mortal Wkly Rep 2009 58 15 400 402 http://review/mmwrhtml/mm5815a5.htm 19390508

[B13] R Development Core Team A language and environment for statistical computing 2010 Vienna: Foundation for Statistical Computing

[B14] World Health Organization Global Alert Response. Situation updates - Pandemic (H1N1) 2009 http://www.who.int/csr/disease/swineflu/updates/en/

[B15] Pada S Tambyah PA Overview/reflections on the 2009 H1N1 pandemic Microbes Infect 2011 May 13 5 470 478 Epub 2011 Jan 27 10.1016/j.micinf.2011.01.009 21276873

[B16] Hartley DM Nelson NP Eli N Perencevich. Antiviral Drugs for Treatment of Patients Infected with Pandemic (H1N1) 2009 Emerg Infect Dis 2000 15 November 2009. http://www.cdc.gov/eid 10.3201/eid1511.090720 PMC2857246 19891884

[B17] Cox NJ Subbarao K Global epidemiology of influenza: past and present Annu Rev Med 2000 51 407 421 Review 10.1146/annurev.med.51.1.407 10774473

[B18] Taubenberger JK Morens DM 1918 Influenza: the mother of all pandemics Emerg Infect Dis 2006 Jan 12 1 15 22 16494711 10.3201/eid1201.050979 PMC3291398

[B19] Chan EH Brewer TF Madoff LC Pollack MP Sonricker AL Keller M Freifeld CC Blench M Mawudeku A Brownstein JS Global capacity for emerging infectious disease detection Proc Natl Acad Sci U S A 2010 Dec 14 107 50 21701 21706 Epub 2010 Nov 29 10.1073/pnas.1006219107 21115835 PMC3003006

[B20] Thomas CS Nelson NP Jahn GC Tianchan N Hartley DM Use of media and public-domain Internet sources for detection and assessment of plant health threats Emerging Health Threats Journal 2011 4 7157 10.3402/ehtj.v4i0.7157 PMC3168368 24149031

[B21] Brownstein JS Freifeld CC Chan EH Keller M Sonricker AL Mekaru SR Buckeridge DL Information technology and global surveillance of cases of 2009 H1N1 influenza N Engl J Med 2010 May 6 362 18 1731 1735 10.1056/NEJMsr1002707 20445186 PMC2922910

